# A mixed methods investigation of implementation barriers and facilitators to a daily mobile phone sexual risk assessment for young women in Soweto, South Africa

**DOI:** 10.1371/journal.pone.0231086

**Published:** 2020-04-23

**Authors:** Janan J. Dietrich, Stefanie Hornschuh, Mamakiri Khunwane, Lerato M. Makhale, Kennedy Otwombe, Cecilia Morgan, Yunda Huang, Maria Lemos, Erica Lazarus, James G. Kublin, Glenda E. Gray, Fatima Laher, Michele Andrasik, on behalf of the HVTN 915 team

**Affiliations:** 1 Perinatal HIV Research Unit (PHRU), Faculty of Health Sciences, University of the Witwatersrand, Johannesburg, Gauteng, South Africa; 2 Health Systems Research Unit, South African Medical Research Council, Cape Town, Western Cape, South Africa; 3 Vaccine and Infectious Disease Division (VIDD), Fred Hutchinson Cancer Research Center, Seattle, WA, United States of America; 4 Office of the President, South African Medical Research Council, Cape Town, Western Cape, South Africa; Centre for Sexual Health & HIV/AIDS Research, ZIMBABWE

## Abstract

**Background:**

The HIV epidemiology in South Africa reveals stark age and gender disparities, with young women being the most vulnerable to HIV acquisition in 2017. Evaluation of HIV exposure is a challenge in HIV prevention research. Intermittent in-clinic interviewer-administered risk behaviour assessments are utilised but may be limited by social desirability and recall biases. We piloted a mobile phone application for daily self-report of sexual risk behaviour in fifty 18–25 year old women at risk of HIV infection enrolled in HIV Vaccine Trials Network 915 (HVTN 915) in Soweto, South Africa. Through a mixed-methods investigation, we explored barriers and facilitators to completing daily mobile phone surveys among HVTN 915 study participants and staff.

**Methods:**

We analysed quantitative data on barriers and facilitators to mobile phone study completion collected during the larger HVTN 915 study as well as two post-study focus group discussions (FGDs) with fifteen former participants with a median age of 24 years (IQR 23–25) and six individual in-depth interviews (IDIs) with HVTN 915 staff. FGDs and IDIs utilised semi-structured interview guides, were audio-recorded, transcribed verbatim and translated to English. After coding, thematic analysis was performed.

**Results:**

The main facilitator for daily mobile phone survey completion assessed across 336 follow-up visits for 49 participants was the daily short message system (SMS) reminders (93%, 312/336). Across 336 visits, 31/49 (63%) retained participants reported barriers to completion of daily mobile phone surveys: forgetting (20%, 12/49), being too busy (19%, 11/49) and the survey being an inconvenience (15%, 9/49). Five main themes were identified during the coding of IDIs and FGDs: (1) facilitators of mobile phone survey completion, such as daily SMS reminders and follow up calls for non-completers; (2) barriers to mobile phone survey completion, including partner, time-related and technical barriers; (3) power of incentives; (4) response bias in providing sensitive information, and (5) recommendations for future mobile phone based interventions.

**Conclusion:**

Despite our enthusiasm to use innovation to optimise sexual risk assessments, technical and practical solutions are required to improve implementation. We recommend further engagement with participants to optimise this approach and to further understand social desirability bias and study incentives in sexual risk reporting.

## Introduction

Nearly four decades into the HIV pandemic, South Africa remains the country with the highest number of citizens living with HIV, with an estimated 13.5% of individuals living with HIV in 2019 [1]. The epidemiology reveals stark age and gender disparities, with young women being the most vulnerable to HIV acquisition in 2017 with an HIV incidence of 1.51 [2]. Innovative approaches to HIV prevention, especially for women in South Africa, are critical.

An integral part of HIV prevention interventions in South Africa, where most HIV transmissions are through heterosexual sex, is understanding sexual risk behaviour [3–6]. However, sexual risk behaviour assessment remains one of the many challenges in HIV prevention research where self-report measures are used [7–10]. Several studies underscore the problems of social desirability bias, recall bias and memory difficulties in self-reported behavioural data [7, 9, 11–14]. Studies show that participants are likely to respond in ways that are culturally and socially acceptable and normative to avoid being judged [9, 12, 13, 15]. Participants may give inaccurate responses to questions due to extended recall periods [16, 17], forgetting or telescoping events they prefer not to remember [11]. Such biases in self-reported data can cause overestimation or underestimation of risk behaviour and affect prevention efforts [11, 18]. There is a need for innovative methods to collect real-time, accurate data on sexual risk behaviour among high-risk populations for HIV.

Mobile health (mHealth) technologies, particularly short messaging services (SMS), are gaining popularity in the HIV field. SMS has been successfully used to improve adherence, provide psychosocial support for people living with HIV and to disseminate HIV-related information [19–23]. Recently, studies have explored the use of mobile phones for sexual and behavioural data collection [24–27]. The most evident advantages for the use of mobile phones in data collection is that they can facilitate data collection in a participant’s preferred location, closer to real-time, and with less social desirability and recall bias [24, 28, 29]. The SMS strategy of data collection has proven to be successful in different geographic regions and among various populations, including people living in peri-urban farming communities in Kenya, young women sharing their abortion stories in Kenya, and high-risk men who have sex with men in the United States [24, 25, 30]. Thus, the sense of privacy and confidentiality provided through the use of SMS services may have minimised reporting bias [24, 30, 31].

Despite the widespread availability of digital HIV prevention interventions for young people in South Africa, we did not find published literature on the use of mobile phone applications (apps) for HIV prevention interventions for young women. In 2018, mobile phone penetration in South Africa was high, with 82% of households owning at least one mobile phone, with smartphone penetration at 51% [32].

In 2015, the HIV Vaccine Trials Network (HVTN) completed a prospective cohort study, HVTN 915, to evaluate the use of once-daily self-administered vaginal swabs over 90 days for the detection of HIV-1 virions among 50 young women in Soweto. [33, 34]. One of the secondary objectives was to pilot the use of a mobile phone survey app to collect the type of sexual activity data that would be applicable to preventive HIV vaccine trials. The mobile phone survey app allowed for the reporting of sexual behaviour outside of the clinical environment (i.e. at their homes or selected venues). Participants self-collected vaginal swabs daily and completed daily mobile phone surveys as well as eight interviewer-administered in-clinic questionnaires over 12 weeks (including the enrolment visit–week 0) to assess sexual risk. As confirmed by detection of the Y chromosome on vaginal swabs, sex acts reported via the mobile phone survey were more accurate than via the in-clinic questionnaire [34]. The results provided evidence that daily mobile phone surveys, with a response rate of 82% (4219/4500 delivered surveys) reduced social desirability bias and recall bias of the clinic-administered behavioural questionnaires which assessed behaviour over a seven day recall period [35]. The objective of the present study was to explore barriers and facilitators to completing daily mobile phone surveys among HVTN 915 participants and staff.

## Methods

**Fig 1:**
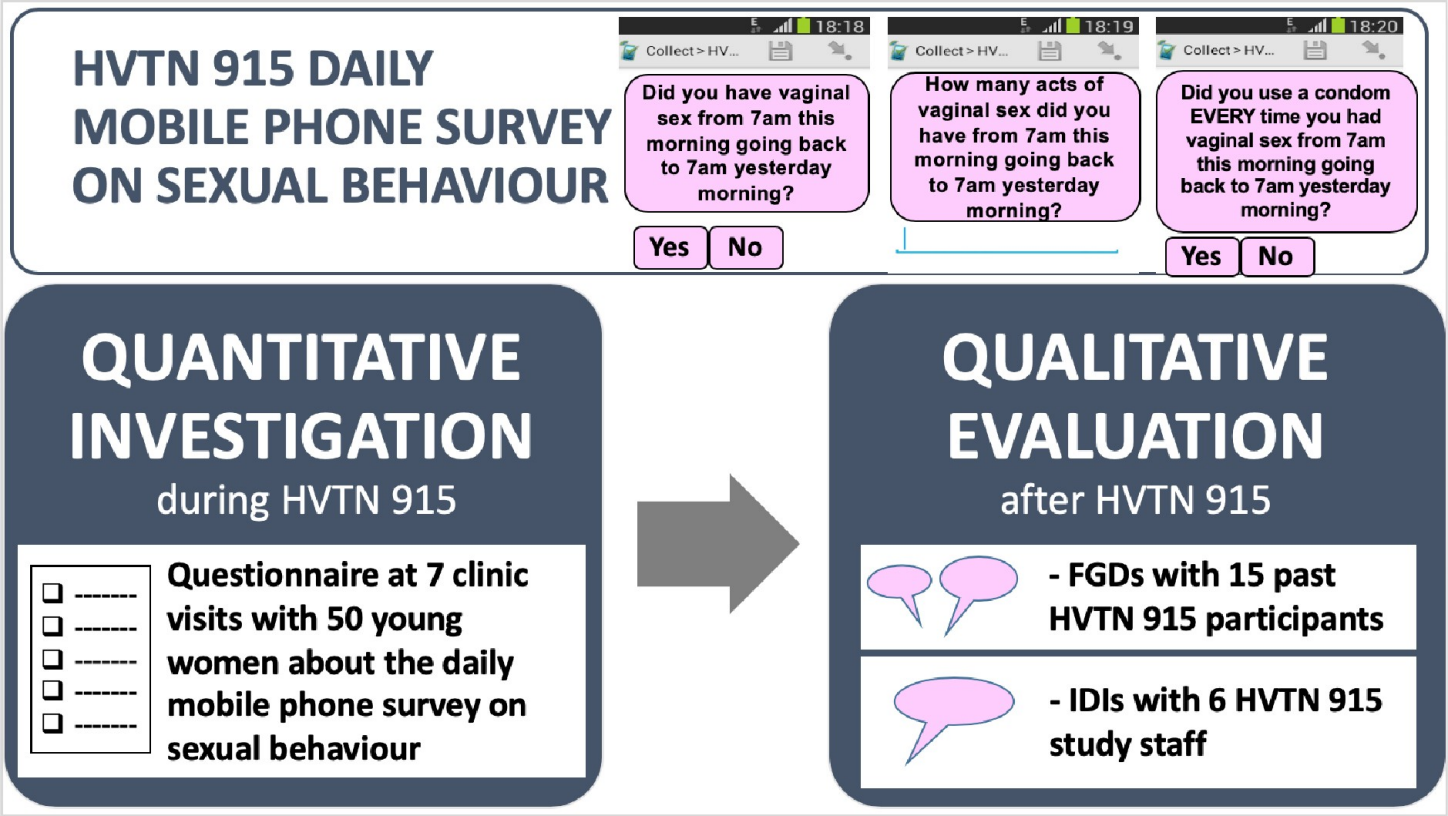
Overview of the quantitative and qualitative evaluations undertaken.

Our study used a sequential mixed methods design (Fig 1). The first component was quantitative data that originated from a structured in-clinic questionnaire administered during the HVTN 915 study. The second was qualitative research conducted post HVTN 915 comprising in-depth interviews (IDIs) conducted with study staff and focus group discussions (FGDs) conducted with participants.

HVTN 915 was a prospective non-intervention cohort study conducted August 2014 to May 2015. Study participants were healthy, heterosexual HIV-uninfected women aged 18–25 years living in Soweto. At enrolment, all women were provided with entry level smartphones (which they could keep at study completion) and sufficient data for survey completion. A participant identity number was required to access the mobile phone survey app. For 12 weeks, 50 young women were expected to complete daily mobile phone surveys assessing sexual risk behaviour. After enrolment, study nurses administered in-clinic questionnaires to assess sexual risk behaviour and mobile phone survey completion during seven scheduled in-clinic visits (i.e. weeks 1, 2, 3, 4, 6, 8 and 12).

We conducted the present study’s qualitative component April to September 2015 (after HVTN 915 concluded). We conducted IDIs with study staff involved in HVTN 915 and facilitated FGDs with HVTN 915 participants to explore barriers and facilitators to completing daily mobile phone surveys.

### Study setting and participants

Both the quantitative and qualitative components were conducted at the Perinatal HIV Research Unit (PHRU) in Soweto, a peri-urban township 15km southwest of Johannesburg, South Africa, where HIV prevalence was 4% among young women 14–24 years [36].

For IDIs, we utilised convenience sampling to select six HVTN 915 staff members: three clinicians (two nurses and one doctor), two counsellors and one mobile phone educator. For FGDs, study staff telephonically invited all 49 participants retained in HVTN 915 to participate. Therefore, a convenience sample of 15 women participated in two FGDs: nine in the first FGD and six in the second.

### Study procedures and measures

#### Mobile phone survey set-up and procedures

We developed the survey using SurveyCTO app, a computer-assisted survey platform with built-in data encryption [37]. Both the SurveyCTO app and survey content were pre-loaded onto the study smartphones. Participants had two levels of passwords: one upon switching on the phone, and the second to enter the SurveyCTO app. The ‘AppLock’ application was installed on each study smartphone to block other internet-enabled apps and ensure that pre-loaded data bundles were used solely for the study survey. Participants required the use of a ‘dash’ (-) function for certain responses, which was not supported by the study phone’s ordinary keyboard. Therefore, the google keyboard was downloaded for participants to enter the dash function when required.

At study enrolment, a mobile phone educator trained participants on both the smartphone and mobile phone survey through a video, practical demonstration, and a brochure. Participants received up to four daily SMS reminders (“It is time”) to complete the five minute surveys (i.e. 6am, 1am, 1pm and 6pm) until the day’s survey was received. Once received, remaining reminders would be cancelled for the day.

The data management company, iKapadata (www.ikapadata.com) [38], generated a daily report for staff to follow up telephonically with participants who had not submitted the mobile phone survey the previous day. Staff provided basic technical support when required and technical support beyond the scope of study staff was referred to iKapadata. After successful study completion, participants could keep the smartphone and AppLock was deleted.

#### Mobile phone survey questions

We used a five-item survey with three questions asking about sexual behaviour [35]. The first question asked if the participant had vaginal sex between 7am on the day [35] of questionnaire completion and 7am the preceding day (24-hour period). If the response to this question was “no”, the survey ended. However, should the participant reply “yes”, then the following two questions were generated: (1) how many acts (“rounds”) of vaginal sex occurred; and (2) whether a condom was used with every vaginal sex act (Fig 1).

*In-clinic questionnaires about demographics and the mobile phone survey*. At the HVTN 915 screening visits, participant demographics, including age and sex at birth, were collected. During clinic visits at weeks 1, 2, 3, 4, 6, 8, and 12, clinicians administered a pen-and-paper questionnaire about mobile phone survey completion. All participants completed questions about facilitators to daily mobile phone survey completion. The lists of facilitators and barriers were developed based on user-testing feedback from the PHRU’s Prevention Community Advisory Board and HVTN 915 staff members. Participants responded to barriers to daily mobile phone survey completion if they self-reported non-completion since the last visit. The non-completion rate across the study period was 18% [35]. Participants selected responses from a pre-specified list of facilitators and barriers to daily mobile phone survey completion. Responses included a 3-point Likert scale (‘never, sometimes, always)’. Facilitators were: receiving daily SMS reminders, short survey completion time, ability to complete the survey at any time and ease of mobile phone use. Participants only responded to the items of barriers if they reported not submitting their daily mobile phone surveys. Barriers were: being too busy, forgetting, inconvenience of completing the mobile phone survey and lack of privacy.

### IDIs and FGDs

Experienced, trained, and multi-lingual interviewers (conversant in English, isiZulu and Sesotho) used a semi-structured guide to conduct IDIs and FGDs in private rooms at the PHRU. Prior to the FGDs, participants completed a brief survey that assessed demographics, mobile phone ownership and use. The IDI and FGD guides explored experiences and challenges of mobile phone survey completion and daily SMS reminders. In addition, discussions elicited recommendations for improving future sexual risk mobile phone surveys. Prior to FGDs, participants were given pseudonyms which they displayed throughout the discussions. Participants were requested to state their pseudonym each time they spoke, which enabled us to ascribe statements to participants. IDIs were conducted in English while FGDs were conducted in a mix of English, isiZulu, and seSotho. The mean time for IDIs was 41 minutes and for FGDs 107 minutes.

Audio recordings of FGDs and IDIs were transcribed verbatim by three multi-lingual transcribers, including LMM. All transcripts were verified by LMM and an additional study team member by listening to the audio-recordings and comparing them with transcripts. Discrepancies were discussed with transcribers and subsequently addressed.

### Data analyses

Statistical analyses for quantitative data were conducted using SAS/STAT software in SAS Enterprise Guide 7.1 (SAS Institute Inc., Cary, NC, USA). Descriptive data including counts and percentages for categorical survey responses and medians and interquartile ranges for numeric responses were determined. We obtained demographic information for HVTN 915 participants through data collected at the screening visits. Mobile phone survey completion was assessed throughout the study.

We utilised inductive and deductive approaches to qualitative data analysis. We analysed the qualitative data through a constant comparison process to identify common and divergent themes and to determine interrelationships between identified themes [39]. We coded all transcripts manually. First, a primary coder (SH) read and re-read all the transcripts to gain an overall understanding of the data and to develop a codebook using an excel spreadsheet. The codebook was developed using a priori approach to identify overall facilitators and barriers to mobile phone survey completion, time and technically-related barriers, response bias in providing sensitive information and recommendations for future mobile phone interventions. After the codebook was developed by the primary coder (SH), two additional coders (LMM, JJD) independently coded all transcripts using the codebook. Addditional codes were added to the codebook. Discrepancies were resolved by discussion. Initial coding involved an open coding method whereby a line-by-line analysis was conducted to assign text to codes. Following open coding, the initial coder started the process of axial coding to understand the relationship between the codes [39]. Codes were then grouped according to categories and summarised as themes and sub-themes.

### Ethical considerations

The University of the Witwatersrand Human Research Ethics Committees (Wits HREC) approved the HVTN 915 study (#131114) and its post-study qualitative component (#H14/11/13). The PHRU HIV prevention community advisory board ratified the relevance of the study design, procedures and mobile phone survey content. Written informed consent, including for audio recording, was obtained from all respondents prior to their participation in the study. HVTN 915 participants received a study phone, ZAR150 (~ USD11) for scheduled study visits, and ZAR5 (~ USD0.36) airtime for each submitted survey. Participants of the post study qualitative component received ZAR50 (~ USD4) reimbursement. At the time of the study, USD1 was equivalent to approximately ZAR14.

## Results

Fifty women from Soweto were enrolled in HVTN 915, attended 336 follow-up clinic visits (excluding screening and enrolment), in total (i.e. approximately 7 out of 9 expected clinic visits per participant). Table 1 presents demographics of FGD (n = 15) participants. The median age for FGD participants was 24 years (IQR 23–25). Overall, 80% (n = 12) of FGD participants spoke IsiZulu as their primary home language, 67% (n = 10) lived in brick houses and up to 47% (n = 7) had not completed high school.

**Table 1:** Demographics, mobile phone usage and internet access characteristics of HVTN 915 FGD participants.

Variable	FGD Participants (n = 15)
**Age**	24 (IQR 21–26)
**Home language**	
IsiZulu	12 (80.0)
Other (IsiXhosa, Sesotho, Setswana)	3 (20.0)
**Dwelling**	
Brick house owned by family	10 (66.7)
Rental flat	1 (6.7)
Shack in an informal settlement	2 (13.3)
Shack in the backyard of a house	2 (13.3)
**Highest level of completed education**	
Completed high school (completed grade 12)	4 (26.7)
Incomplete high school	7 (46.7)
Incomplete post-high school training	4 (26.7)
**Do you own or share a personal mobile phone?**	
Yes	15 (100)
**How much time in a day do you spend actively using a mobile phone?**	
0–1 hour	2 (13.3)
2–4 hours	3 (20.0)
5–7 hours	2 (13.3)
More than 8 hours	4 (26.7)
Don't know	4 (26.7)
**How do you get airtime?**	
Prepaid	15 (100)
**Use mobile phone to make and receive calls**	11 (73.3)
**Use mobile phone to send and receive SMS**	11 (73.3)
**Use mobile phone for playing games**	4 (26.7)
**Use mobile phone to access the internet**	8 (53.3)
**Use mobile phone to access social networking Sites (i.e. Facebook, Twitter, Instagram)**	7 (46.7)
**Use mobile phone to access WhatsApp**	8 (53.3)
**Access to the internet in the last 6 months**	14 (93.3)
**Finding health information through the internet**	5 (33.3)
**Visit social media sites through the internet**	9 (60.0)

### Mobile phone ownership and use

All FGD participants (n = 15) owned a mobile phone of which 67% (n = 10) owned a smart phone and 13% (n = 2) shared a personal mobile phone. All FGD participants used pre-paid airtime, 20% (n = 3) used their mobile phones 2–4 hours daily and 27% (n = 4) used them for more than 8 hours per day. Participants used their mobile phones as follows: 73% (n = 11) making and receiving calls and for sending and receiving SMSs, 27% (n = 4) playing games, 53% (n = 8) accessing the internet, 47% (n = 7) accessing social networking sites (i.e. Facebook, Twitter, Instagram) and 53% (n = 8) accessing WhatsApp. In the last six months (to time of FGD and IDI participation), 93% (n = 14) had accessed the internet via a mobile phone or another device (i.e. tablet, laptop or computer), of which 33% (n = 5) reportedly searched for health information and 60% (n = 9) visited social media sites (Table 1).

### Facilitators of mobile phone survey completion

The main facilitator for completing the mobile phone survey was the daily SMS reminders (93%, 312/336). Additional facilitators included: the ease of using the mobile phone (44%, 149/336), and the fact that the survey was easy to complete (30%, 101/336) and the ease of answering the questions (30%, 99/336) (Table 2). Most FGD and IDI participants stated that the daily automated SMS reminders and follow-up calls for survey non-completers were helpful. Overall, FGD participants stated that the mobile phone survey questions were easy to understand in English, they were comfortable with the brief five-item mobile phone survey and these questions were easy to answer. Study staff agreed that mobile phone questions were simple, short, and brief. Two FGD participants stated however that the mobile phone survey question, “Did you use a condom EVERY time you had vaginal sex from 7am this morning going back to 7 am yesterday morning?” was confusing.

**Table 2:** In-clinic nurse-administered questionnaire to assess facilitators for the mobile phone survey across 336 follow-up visits (n = 50 HVTN 915 participants).

Variable	Number (%)
**Did daily reminder SMS help you complete the mobile phone questions?**	
Yes	312 (92.9)
No	24 (7.1)
**Did the fact that it was fast to do help you to complete the mobile phone questions?**	
Yes	101 (30.1)
No	235 (69.9)
**Did the ability to fill out the mobile phone questions anytime help you to complete them?**	
Yes	59 (17.6)
No	277 (82.4)
**Did the ease of using the mobile phone help you to complete the mobile phone questions?**	
Yes	149 (44.3)
No	187 (55.7)
**Did the ease of answering the questions help you to complete the mobile phone questions?**	
Yes	99 (29.5)
No	237 (70.5)
**Did using a study phone instead of your own phone help you to complete the mobile phone questions?**	
Yes	49 (14.6)
No	287 (85.4)

Generally, HVTN 915 participants reported to study staff that the study mobile phone touch screen was easy to use, even though some participants did not own a smartphone themselves. FGD participants and study staff gave examples of additional strategies participants used to remind themselves about mobile phone survey completion, including setting an alarm or incorporating survey completion during their wake-up and morning routine. For example, a study staff member stated:

“They [HVTN 915 participants] come up with their own strategies…some of them would say: ‘I normally wake up in the morning, and take a bath or [my] … small child who attends a day-care centre; so every morning when I wake up that would be the time when I’d be reminded, or [every morning] I brush my teeth…' So, I would help them [HVTN 915 participants]… to come up with the decision whether that would work for them.”

A FGD participant further explained:

“Because in the morning is when there is time but sometimes when you’re lazy to wake up… you know that the phone is nearby, everything is, um, in the morning you’re able to do it.”

### Barriers to mobile phone survey completion

Across the 336 scheduled participants follow-up visits, there were 59 (17%) clinic visits where 31 women reported not submitting their daily mobile phone surveys. These women reported that barriers for non-completion were: forgetting (20%, 12/59), being too busy (19%, 11/59) and the survey being an inconvenience (15%, 9/59) (Table 3).

**Table 3:** In-clinic nurse-administered questionnaire to assess barriers for mobile phone survey non-completion across 59/336* follow-up visits (n = 31 HVTN 915 participants).

Variable	Number (%)
**How often did you not complete because you were too busy?**	
Always	0 (0)
Never	48/59 (81.4)
Sometimes	11/59 (18.6)
**How often did you not complete because you forgot?**	
Always	2/59 (3.4)
Never	45/59 (76.2)
Sometimes	12 /59 (20.3)
**How often did you not complete because it was inconvenient?**	
Always	0 (0.0)
Never	50/59 (84.7)
Sometimes	9/59 (15.3)
**How often did you not complete because you didn’t have privacy?**	
Always	0 (0.0)
Never	57/59 (96.6)
Sometimes	2/59 (3.4)
**How often did you not complete because you were worried your partner would find out?**	
Always	0 (0.0)
Never	57/59 (96.6)
Sometimes	2/59 (3.4)
**How often did you not complete because you misplaced the cellphone?**	
Always	1/59 (1.7)
Never	54/59 (91.5)
Sometimes	4/59 (6.8)

*There were 59/ 336 (17%) scheduled clinic visits (excluding screening and enrolment) where participants reported mobile phone survey non-completion.

Overall, FGD participants stated that they found it challenging having a study mobile phone in addition to their personal mobile phone. However, some FGD participants stated that the study mobile phone served as a back-up contact number. Three FGD participants admitted that they regularly forgot their study mobile phone at home and only carried their personal phone with them because of convenience. For some FGD participants, the study phone became “boring” because the AppLock application restricted its use to the survey functionality.

### Living environment as a barrier to mobile phone survey completion

Study staff reported that participants hid the study mobile phone if they lived with partners or family members who used drugs. In this context, participants completed their mobile phone surveys when the partners or family members were not around. A study staff member explained:

“Some of them [HVTN 915 participants] had boyfriends that smoked *nyaope* [type of local drug]. Some of them used to live with their main partner, they were afraid…that if the partner would see the cellphone they would take the cellphone and sell it.”

A FGD participant told her experience:

“But another thing, my phone was stolen by my drug-addict (brother/cousin), like… he went to get it where I usually get it charged and said that I had sent him to pick it up… then he took it like that. Gone, till today I haven’t seen him.”

Very few participants stated poor housing conditions as a barrier to survey completion. Instead, study staff indicated that lack of privacy was a barrier, especially for participants who had not disclosed study participation to their household members. Study staff expected participants who lived in households with many visitors or those who lived in poorer housing conditions (e.g. not having regular access to electricity to charge the mobile phone) not to complete surveys frequently. However, study staff reported that these types of participants were surprisingly adherent and creative in ensuring they could complete the daily surveys.

A study staff member stated:

“One [HVTN915 participant] told me that…when her phone is flat, she would go to a friend and she’d stay there until the phone is full [charged]. Then she’d take her phone back… And some would actually come here to the site and actually ask us to charge their phones.”

A FGD participant explained:

“Where I stay there isn’t any electricity… so, when I noticed that next door they had put on their generator, I remembered that I had asked them to charge it.”

### Time-related barriers

Study staff reported that many HVTN 915 participants attended school or work, with hours that were long and sometimes irregular, creating difficulties with submission of daily surveys over a long period of time. Study staff also reported difficulties telephonically contacting some participants who did not complete surveys.

Some participants had low rates of survey completion after a weekend. Reasons given to study staff included being away from home for the weekend or ‘feeling lazy’ to complete the survey after a long night out. One participant stated during the FGD that she regularly forgot to complete the survey over the weekends, but that it was easy for her to complete the survey on weekdays:

“I would just forget on the weekend, because I was always drunk… But I know that when I leave on Friday, I’m done, I’ve done them [completing the survey].”

A study staff member reported that one participant was unable to complete the survey due to her child’s sickness as she had spent the day at the hospital. One school-going participant’s study phone was confiscated by the teacher and the principal imposed a cash penalty; study staff negotiated its retrieval.

### Technical barriers

Technical barriers were predominantly reflected in study staff responses. These barriers included: inadvertent disabling of the mobile phone to the network connection resulting in the inability to transmit the completed mobile phone surveys to the study data server, receiving SMS reminders after a successful survey submission, delays receiving data bundles on the study mobile phone, unintentional disabling of the google keyboard ‘dash’ function, re-setting the study mobile phone, disrupting the survey application by charging the mobile phone via a computer or taking the Subscriber Identification Module (SIM) card out of the study mobile phone.

“Well what we didn’t anticipate is that when you have a prepaid card and you load data, the data expires after one month… so we had one or two cases where the data wasn’t sent in time… because they had server problems over the weekend or something.”

Many participants unintentionally disabled the network function on the study mobile phone, which resulted in a chain of technical issues. The completed survey was then automatically saved in the outbox of the survey application, but was not sent to the database server. Consequently, participants did not receive their daily airtime incentive and continued to receive SMS reminders to submit their surveys.

Often, participants re-submitted the same survey several times on the same day. When the network was re-enabled, this resulted in multiple surveys received for the same participant for the same day but often with data inconsistencies between them. Even after study staff verified with the participants the valid survey record for the day and re-trained those participants, the same problem persisted.

One staff member explained:

“…Others would say… I’m trying to send… surveys, but no response. Maybe they thought if they sent that [multiple surveys throughout the day]… they would receive airtime… or maybe… so that they can be sure that we received their surveys.”

FGD participants reported similar technical issues with continuing to receive SMS reminders even after successfully submitting their daily survey. The SMS reminders were intended to be sent until participants completed the survey for the day, so it was confusing for them to receive an SMS reminder when they thought they had already submitted the survey.

One FGD participant described her experience:

“Those SMS’s were annoying… even if you’d done them [survey]… they should find another way of remind[ing] us… when you’d received the first SMS… still 10 times!”

Another technical barrier comprised the automated data bundles issued to study mobile phones. In South Africa, data bundles expire after a month and were therefore uploaded onto the study mobile phones monthly by iKapadata. However, study staff reported that in some instances, there were delays in receipt of data bundles, which affected participants who were unable to submit surveys until the data bundles were received.

Study staff also pointed out that occasionally, the google keyboard function was disabled by the phone itself or the participant disabled the function and the dash on the keyboard disappeared, resulting in some participants not being able to enter the correct participant identity number format and not being able to submit the survey. Study staff did not always feel equipped to trouble shoot technical challenges. One study staff member explained:

“…The one-time… I think it was her [participant’s] brother, he got hold of the phone and he erased the whole content on the phone … that means that I had to…set up the phone here at clinic again which was very challenging…”

Other participants charged their study mobile phone via a computer or took the SIM card out of the study phone, which ‘crashed’ (disrupted) the survey application. In both instances, the survey had to be manually downloaded by the dedicated staff members on site in consultation with off-site database managers.

### The power of incentives

Both study staff and FGD participants stated that cash, airtime and being able to keep the study mobile phone post-study were motivators to join and stay in the study. The automated airtime incentives were a major motivator to complete the survey daily. One FGD participant said:

“I have to go and do them [surveys], because I need that airtime….”

Study staff began questioning whether some volunteers who came to the site to be screened for the study were providing accurate information at screening so that they could sway the study staff’s eligibility assessment. A staff member said:

“A lot of them [potential volunteers] had contrived their stories to meet the eligibility requirements, to get on the study for the purpose of the phone… they were horribly distressed when we told them that they couldn’t be [on the study]”

A FGD participant stated that the study mobile phone and study visit reimbursement were the main reasons for her to join the study:

“Let’s just all tell the truth; we were here for the phones… they’d [study staff] tell me, come with x number of people… and I’ll come with those… and I’ll tell her that, friend, you’ll get a phone and ZAR150 [~ USD11]; and obviously she will come, she wants it.”

FGD participants reported that for many women, the study visit reimbursement was an interim main source of income to cover living expenses for themselves and their families. A FGD participant explained:

“I know I am going to get ZAR150 [~USD11]… I am covered for the week… as long as I receive that ZAR150. It helps me, I live in a township.”

### Response bias in providing sensitive information

Six FGD participants admitted response bias in reporting about their sexual partners and behaviour. Their perception of being eligible for study participation was that they had to report having multiple partners and frequent sexual activity. They also admitted to sometimes over reporting sexual activity in the mobile phone surveys and in-clinic questionnaires; with some confessing that they mainly had one sexual partner throughout the study. One FGD participant said:

“They’ll ask you how many times did you have sex with your casual partner. There’s no casual partner, I only had sex with one person. But because you don’t want to say one person, you’ll fabricate.”

She further reported that she made up responses, which were different during each in-clinic questionnaire:

“Like don’t they take your file and check how accurate what you really say is? Because at times I would forget, you’d find what I said last week, when I get there today, I say something else; my story isn’t coherent, it’s not the same.”

Conversely, staff members reported that some participants had the impression that study staff knew their survey responses, which was a motivator to mobile phone survey submissions and to answer questions truthfully.

Some FGD participants complained that the same questions they had answered daily through the mobile phone survey were also asked during the in-clinic surveys. A FGD participant explained:

“I answered the [mobile phone] survey didn’t I? The same things I answered in the [mobile phone] survey you’re asking me again when I get there [in-clinic study visit]. Then when I lie on the [mobile phone] survey and then I forget, and I say something else at the clinic.”

Once participants were familiar with the mobile phone questions, it was easier to make up responses and finalise the survey quickly. One FGD participant said:

“You started lying, because for the first week you were not lying, you were not comfortable…[We] got used to the lying, you know, after this question, it’s these questions.”

Although some FGD participants stated over reporting sexual activity in the mobile phone survey when they had not had sex, they stated answering honestly when they actually had sexual intercourse. One FGD participant explained:

“Like you had sex honestly, this time, and then you’ll tell yourself you should do the survey. So when it happens that [you] have done it [sex], it becomes I have the time and I had sex, how many rounds 3 times.”

However, some study staff stated that they felt that the majority of participants were honest in answering the mobile phone surveys. A study staff member said:

“So I think that’s one of the successes that they were motivated enough and they did it and we can say we can try trust the data.”

### Recommendations for future mobile phone interventions

Most study staff members recommended that participants received study phones for future studies. Study staff reported on potential challenges if participants were to complete the survey on their personal mobile phone, and recommended using a study mobile phone to ensure a sense of ownership and responsibility. One study staff member stated:

“They change phones frequently… that’s a hallmark of cellphone use in this country… we have that problem constantly because we try to contact participants… and they’ve changed their numbers.”

Study staff further suggested that one staff member should be dedicated to the mobile phone component for the study duration. They reported that it was time-consuming to resolve technical barriers and to follow up on participants who had not completed the daily mobile phone surveys. Staff recommended minimising technology-related barriers, such as dash keyboard difficulties, avoiding the submission of multiple daily surveys, and ensuring that airtime incentives were seamlessly automated. One FGD participant suggested that a variety of content options for the SMS reminders should be provided rather than sending the same message every time up to four times a day (i.e. “It is time”).

Both, study staff and FGD participants suggested alternative methods for sexual risk data collection. For example, social media platforms, messenger apps, a webpage linked to a Facebook page providing sexual health information, and a chat platform app like WhatsApp that are easily accessible to young people, convenient and cost-effective. FGD participants perceived that people would be more likely to respond to a WhatsApp message than a SMS, as it was cheaper to buy a data bundle rather than a SMS bundle for a small of amount of money and more messages could be sent. FGD participants also suggested a game app, pre-loaded on the phone, with different levels and challenges, as another way to collect information, including an option for participants to ask questions.

One FGD participant explained that using SMS or using an app to answer questions is an anonymous way to collect information and participants do not have to disclose their participation. However, asking questions through a phone call or using phone calls to remind the participant about answering questions may make the partner suspicious. She said:

“You’re calling and you’re calling out numbers like he’ll eventually come to ask you why is this person calling you every day…and you cannot explain that they’re calling me because there’s a survey.”

Participants suggested that the easiest way to log an answer would be by using pre-populated touch screen options, if collecting information through a mobile app, rather than manually entering a response with the touch screen mobile phone keyboard.

## Discussion

The present study provides critical insights from participants and study staff about the collection of self-reported sexual risk information via a mobile phone application platform. To our knowledge, this is one of the first studies to incorporate daily HIV risk assessment questions delivered via mobile phones. For this innovation to be taken up. we need to ensure that all technical and practical barriers are addressed.

For researchers, it seemed the main facilitator for participants completing the mobile phone survey was the ease of using a mobile phone and/or survey app. This was evident in the high response rate to the daily mobile phone survey over the 3 month period (82%), which also demonstrated that more sex acts were reported by the HVTN 915 participants through the mobile phone survey, compared to in-clinic interviewer-administered surveys [33, 35]. The response rate of 82% was similar when compared with other, comparable mobile phone surveys, which was deemed as high. Those studies measured sexual risk behaviour and mental health amongst different populations globally and HIV risk amongst female sex workers in the US and response rates ranged between 80–90% [26, 40, 41]. Studies from other low and middle income countries, like Bangladesh and India [42–45], confirmed similar observations, that the strengths of using mobile phone surveys to collect health information included more accurate responses to survey questions [24, 28, 29, 46]. This must also be weighed against the fact that some participants indicated to have provided misinformation about sex acts, which may have artificially increased their reported risk. Over reporting of non-existent sexual behaviours also assists in understanding results from the larger study that found fewer HIV exposures than expected [46].

For participants of our qualitative study, it was advantageous that the survey only had a few questions, reducing respondent fatigue [47]. In our study we found that most HVTN 915 participants reported quantitatively that their main facilitator in completing the daily mobile phone surveys were the daily SMS reminders. However, during FGDs women reported that the same messaging content was bland and “annoying” over a long period of time and that sometimes SMSs were still received after a submitted survey record. In future, SMS reminders could be programmed in a more individualised and responsive way, to allow participants to select reminder frequency, content and timing, with reminder messages stopped once a participant has submitted their survey.

As in other mHealth studies, technical challenges can pose a barrier to mobile phone survey completion [43–45, 48]. As mobile technology is incorporated into health research and programmes, the new world of work may require shifting skill sets for the next generation of researchers and implementers. Researchers will have to test various technological approaches to incorporate in-clinical trial operations for both participants and study staff.

The power of incentives was a theme that influenced participation and reporting in the larger HVTN 915 study. Participants reported that the multiple incentives influenced their willingness to join the overall HVTN 915 study and to complete the study procedures. Some standard benefits for many research trial participants are visit reimbursements and ancillary health care access [49]. Many who take part in HIV research are often from communities with high levels of unemployment [50] and research participation may provide an income source [51]. Participants in other mHealth research have also received incentives [48, 52–54]. In our study, there was also an airtime and a mobile phone benefit. Participants reported the multiple study incentives as a motivator, for some the study visit reimbursements were an interim source of income. In addition, a few participants who participated in the FGDs reported that they provided inaccurate information about their sexual behaviours to participate in the study and receive the benefits. However, these data on providing inaccurate data must be understood within the context of the larger study that assessed behavioural data with seminal markers [35, 46]. There was relatively accurate reporting of sexual activity with the daily phone compared with in-clinic sexual risk reporting.

Six of the women stated over reporting sexual activity because of social desirability bias. A study in rural Kwa-Zulu Natal showed that participants were more likely to report “socially undesirable sexual behaviours”, such as the number of sexual partners in the last year or one’s history of anal sex, via electronic delivery methods [48]. In another study it was found that a person’s age, personality and gender were either barriers or facilitators in aiding participants to provide timely, accurate and undesirable sexual behaviour [9]. Some participants in our study stated providing inconsistent information on both the in-clinic and mobile survey. However, they reported that when sexual activity actually occurred, they reported more accurately via the mobile survey. Their report of sexual activity can be linked to their actual sexual activity using the mobile phone survey [35]. For future studies, it would be important to determine whether response bias is indeed reduced with an event based mobile phone survey instead of daily reporting.

Some women reported difficulties disclosing study participation to their partner and/or household family members who used drugs, having to hide their study phone. There is evidence from other studies in sub-Saharan Africa that male sexual partners influence women’s ability and willingness to join HIV prevention studies [55]. Women’s disclosure of study participation or lack thereof may affect their behaviour and adherence to study procedures [56]. Even though some women in our study were not able to disclose study participation, they were overall adherent to study procedures as shown by the high rates to survey completions and openly communicated to study staff when there were instances they were not able to complete study procedures.

With conventional (for example, male condoms, female condoms, PrEP) prevention strategies, the tide of HIV incidence is turning in many age and gender demographics in South Africa, but young women remain an exception [2]. Engaging young women in health research and interventions is a challenge requiring special consideration and possibly non-conventional strategies. Participants in our study suggested more entertaining technological approaches to stay relevant to their demographic, such as the use of gaming and social networking applications to collect sexual risk information. Most social networking sites are not private or secure, and due consideration would need to be given to balance the need for privacy and security of participant data with the preference for young people to interact through accessible technology [57].

### Limitations

Qualitative data must be understood within the context of the women who participated in the FGDs but who do not represent the responses of all of the HVTN 915 participants. Our qualitative findings are limited to the FGD participants aged 21–26 years. Therefore, the views of the participants aged 18–20 years were not represented. A limitation of the present study is the small sample size for the qualitative component. Therefore, the qualitative findings cannot be generalised to all HVTN 915 participants. Further, Soweto is a community where several research studies and trials have taken place (especially for young women). Therefore, it is likely that many communities know PHRU which could have influenced participants' willingness to participate in the larger HVTN 915 study. Although our mixed methods study drew upon a small sample of the original HVTN 915 study, our study design has inherent strengths in that we attempted data triangulation through including quantitative data from HVTN 915 participants’ in-clinic visits, and qualitative data from a subset of HVTN 915 participants’ and study staff perceptions and experiences. In addition, participants only reported barriers during the in-clinic survey if they indicated that they did not submit their mobile phone surveys. Hence, the majority of participants would have not reported barriers to the mobile phone survey completion because the overall mobile phone survey response rate was high (85%) [35].

## Conclusion

Despite our enthusiasm to use innovation to optimise sexual risk assessments, technical and practical solutions are required to improve implementation. We recommend further engagement with participants to optimise this approach and to further understand social desirability bias and study incentives in sexual risk reporting. More user-engaged technical development and testing among different populations is required to optimise this approach to achieve more widespread acceptance and use in HIV prevention clinical trials.

## Supporting information

S1 File Interview and focus group discussion guides. (DOC)

S2 File (PDF)
