# Supplementary material for: A mixed methods investigation of implementation barriers and facilitators to a daily mobile phone sexual risk assessment for young women in Soweto, South Africa
Source: PLoS One. 2020 Apr 23;15(4):e0231086. doi: 10.1371/journal.pone.0231086 (PMC7179867; doi:10.1371/journal.pone.0231086)
Supplement: S1 File — (DOC) [file pone.0231086.s001.doc]

**Interview Guide for Focus Group Discussions and In-depth Interviews**

**Title:** User-friendly technologies for HIV prevention trials. Using a mobile phone messaging platform for sexual risk assessments among young women in Soweto, South Africa

**Investigators**

Dr. Janan Dietrich, Stefanie Hornschuh, Celokhule Tshabalala, Gugulethu Tshabalala, Lerato Mkhale, Lethabo Ramskin

**Note: This is a semi-structured interview guide which may change based on further literature searches, focus group discussions and in-depth interview feedback.**

**Part 1**

**For both groups of women**

**Let’s start with talking about mobile phones and how we can use them to collect information.**

1. What do you and your friends usually use your phones for *(Probe: send and receive SMS, phone calls, listening to music, play games, access the internet, Whats app, You tube etc.)*
2. What happens if you do not have a phone? Whose phone can you use? What are some of the disadvantages of using someone else’s phone?
3. - What type of phones do young women in your community have?

- What apps do they use?
- If they go onto the internet, what are they searching for? *(Probe: Downloading music, research for project, dating sites, social networking sites, to find information about relationships, to find information about health ,e-mail, internet banking, selling and buying sites etc.)*

1. How would you develop a SMS or mobile phone programme that asks for information about your romantic relationships?

*Prompts: What would be the content, in which language would you formulate the messages (English or home language), would you use formal/informal content (e.g. modern language, such as LOL, smileys?)*

1. What are some of the issues/problems we have to think about in developing a mobile phone programme?

*Prompts: Does the majority has internet access on their mobile phones? Risk of others seeing sensitive topics while participating in a study, what time should messages be sent out (e.g. in the morning, afternoon, evening?)*

1. How can we approach young women that are at high-risk for HIV to take part in a mobile phone programme? How can we include young women who do not have their own mobile phones? How can we include women who do not have access to a mobile phone?

**For women who did not test the mobile phone messaging platform**

1. We realize that many phones that people have in Soweto are not internet enabled, in some studies we would be able to give people a phone. How would you feel having two phones with you (your own and study phone)? Would it be easy or difficult to look after two phones and to carry them with you all the time? What are the pros and cons*?*

**Part 2**

**For women who participated in the study testing the mobile phone messaging platform only**

**Now we are going to talk about your experiences with the mobile phone survey and vaginal swab that you have completed over the period of 3-month.**

1. What were the challenges in your lives that impacted your ability to complete study procedures (i.e. survey, swabs) after enrolling in the study? E.g. work, relationship problems, housing, privacy issues.
2. **Vaginal swabs:**

What were the challenges and successes for you when collecting your daily swabs?

*(Probes: What were the situations where they were not able to do their swabs? What strategies did help to be able to do the swabs?)*

1. Tell me about your experiences with the survey application and the smart phone that we provided to you. Was it easy to use? Why,why not? What did you like about taking part in the study? What did you not like?
2. You filled in very sensitive information about your sexual activity. How truthful were you about the information that you gave us? What were the challenges?
3. What would you make better for future mobile phone surveys? *(Probe: Amount of questions, the way the questions were asked, language (English or home language), ideas for design appearance (e.g. colours, pictures, set up).*
4. What did you think of the survey app?
5. Should we use the same messaging platform that works via the internet?Why/why not?
6. Did the SMS reminders help you to complete your survey?
7. What are others ways on how we can collect sexual risk data via a mobile phone? *(Probe: SMS, games etc.)*
8. We would like to find out on how we can improve future studies that collect sensitive behavioural data. Should we provide women again with a study phone or should they use their personal phone? What are the pros and cons for using a study phone/personal phone?
