# Supplementary material for: A mixed methods investigation of implementation barriers and facilitators to a daily mobile phone sexual risk assessment for young women in Soweto, South Africa
Source: PLoS One. 2020 Apr 23;15(4):e0231086. doi: 10.1371/journal.pone.0231086 (PMC7179867; doi:10.1371/journal.pone.0231086)
Supplement: S2 File — (PDF) [file pone.0231086.s002.pdf]

Study Number ☐ ☐

DEMOGRAPHIC QUESTIONNAIRE

Personal Information

Age ______ yrs

What is the Area/Township that you live in: ________________________________

What is your primary (main) home language:

Afrikaans	☐	IsiZulu	☐	SiSwati	☐
English	☐	Northern Sotho	☐	Tshivenda	☐
IsiNdebele	☐	Sesotho	☐	Xitsonga	☐
IsiXhosa	☐	Setswana	☐		

Other (please specify) __________

What is the main material that the walls of your house are built of?

- ☐ Brick house owned by family
- ☐ Brick house that family is renting
- ☐ Flat owed by family
- ☐ Flat that family is renting
- ☐ RDP house
- ☐ Hostel (Brick)
- ☐ Shack - Informal settlement
- ☐ Shack – Backyard

Other, Specify ____________________

Study Number ☐ ☐

What is the highest level of formal education you have completed? *Please select one.*

- ☐ No formal education
- ☐ Incomplete primary school (up to grade 7)
- ☐ Complete primary school (completed grade 7)
- ☐ Incomplete high school (up to grade 12)
- ☐ Complete high school (completed grade 12)
- ☐ Incomplete post-high school training (Trade or technical training, college, or university)
- ☐ Complete post-high school training (Trade or technical training, college, or university)
- ☐ Other (*please specify*): ______________________

Mobile Phone Usage

Do you own a personal mobile phone?

☐ Yes
☐ No

Do you share your personal phone with someone?

☐ Yes
☐ No

What type of phone (model) is it?

Please specify: ________________________________

☐ Don't know

What do you use the mobile phone for? *Please read list and select all that apply.*

- ❑ Send and receive SMS
- ❑ Calling
- ❑ For emergencies only
- ❑ Making phone calls

Study Number ☐ ☐

- ☐ Listening to music/radio
- ☐ Play games
- ☐ Access the internet
- ☐ Mxit
- ☐ Facebook
- ☐ Twitter
- ☐ Whatsapp
- ☐ BBM
- ☐ We Chat
- ☐ Youtube
- ☐ Cellphone banking
- ☐ Instagram
- ☐ Toilet
- ☐ 2 Go
- ☐ E-mail
- ☐ Other (*please specify*): ______________________

How much time in a day do you spend actively using a mobile phone? *This includes using it for listening to music/radio, SMS, making phone calls, playing games and accessing the internet.*

- ☐ 0-1 hours
- ☐ 2-4 hours
- ☐ 5-7 hours
- ☐ More than 8 hours
- ☐ Don't know

How to you get airtime?

- ☐ Prepaid
- ☐ Contract
- ☐ Don't know

Study Number ☐ ☐

Internet Access and Usage

In the last 6 months, have you had access to the internet?

- ❑ Yes
- ❑ No

When you are on the internet what do you do or search for? *Please read list and select all that apply.*

- ❑ Download music and videos
- ❑ Research for school projects
- ❑ Dating sites
- ❑ To get directions to places (GPS) and look at maps
- ❑ To find information about health
- ❑ To find information about romantic relationships
- ❑ To find out about parties/ DJ events
- ❑ Internet banking
- ❑ To use social media sites (e.g., Mxit, facebook, etc.)
- ❑ E-mail
- ❑ Selling and buying sites (e.g. Olx, GumTree or Bid or Buy)
- ❑ Other (please specify): ______________________

Are you on Facebook?

- ❑ Yes
- ❑ No

Do you have an email address?

- ❑ Yes
- ❑ No
